# Heritable Stochastic Switching Revealed by Single-Cell Genealogy

**DOI:** 10.1371/journal.pbio.0050239

**Published:** 2007-09-04

**Authors:** Benjamin B Kaufmann, Qiong Yang, Jerome T Mettetal, Alexander van Oudenaarden

**Affiliations:** 1 Department of Physics, Massachusetts Institute of Technology, Cambridge, Massachusetts, United States of America; 2 Division of Engineering and Applied Sciences, Harvard University, Cambridge, Massachusetts, United States of America; Institute for Information Transmission Problems, Russian Federation

## Abstract

The partitioning and subsequent inheritance of cellular factors like proteins and RNAs is a ubiquitous feature of cell division. However, direct quantitative measures of how such nongenetic inheritance affects subsequent changes in gene expression have been lacking. We tracked families of the yeast Saccharomyces cerevisiae as they switch between two semi-stable epigenetic states. We found that long after two cells have divided, they continued to switch in a synchronized manner, whereas individual cells have exponentially distributed switching times. By comparing these results to a Poisson process, we show that the time evolution of an epigenetic state depends initially on inherited factors, with stochastic processes requiring several generations to decorrelate closely related cells. Finally, a simple stochastic model demonstrates that a single fluctuating regulatory protein that is synthesized in large bursts can explain the bulk of our results.

## Author Summary

When cells divide, not only DNA but an entire pattern of gene expression can be passed from mother to daughter cell. Once cell division is complete, random processes cause this pattern to change, with closely related cells growing less similar over time. We measured inheritance of a dynamic gene-expression state in single yeast cells. We used an engineered network where individual cells switch between two semi-stable states (ON and OFF), even in a constant environment. Several generations after cells have physically separated, many pairs of closely related cells switch in near synchrony. We quantified this effect by measuring how likely a mother cell is to have switched given that the daughter cell has already switched. This yields a conditional probability distribution that is very different from the exponential one found in the entire population of switching cells. We measured the extent to which this correlation between switching cells persists by comparing our results with a model Poisson process. Together, these findings demonstrate the inheritance of a dynamic gene expression state whose post-division changes include both random factors arising from noise as well as correlated factors that originate in two related cells' shared history. Finally, we constructed a model that demonstrates that our major findings can be explained by burst-like fluctuations in the levels of a single regulatory protein.

## Abstract 3

When cells divide, each daughter cell inherits a share of the contents of the mother. If the contents include a regulatory system with a feedback loop, sister cells switch states in synchrony.

## Introduction

Inheritance is more than the faithful copying and partitioning of genomic information. When cells divide, the mother cell passes numerous other cellular components to the freshly born daughter, including nucleosomes, transcription factors, mitochondria, and substantial fractions of its proteome and transcriptome. In this way, an entire pattern of gene expression can be passed from mother to daughter, a phenomenon known as epigenetic or non-Mendelian inheritance. Classic examples permeate the literature and include the sex-ratio disorder in *Drosophila* [1], the yellow-tip phenotype in melons [2], the telomere position effect in yeast [3] and mouse [4], and prions such as Psi+ in yeast [5].

The time scale over which epigenetic phenotypes may persist spans many orders of magnitude and depends strongly on the physical mechanism used by the cell [6]. In general, however, epigenetic phenotypes are substantially less stable than chromosomally inherited ones are [6,7], and can change reversibly in single cells [3,8,9] during development [10,11], or in mature organisms [12].

Beginning with landmark studies on the *lac* operon in the 1950s, positive transcriptional feedback loops have emerged as a means to store cellular memory [13–15]. Such epigenetic inheritance systems are frequently described as “bistable,” meaning that transcriptional activity of genes in the network tends to become fixed in single cells around one of two stable levels (ON and OFF), each of which is able to stably persist for many generations [8,16,17]. Stochastic fluctuations in the creation or decay of the proteins involved [18–34], or changes in external cues (e.g., a changing environment), are responsible for causing transitions between the two states [8,13,16,17].

This flexible strategy, which is present in both prokaryotes and eukaryotes, allows genetically identical cells to diversify their population, possibly allowing them to exploit new environmental niches or to survive in a fluctuating external environment [35]. Feedback-based cellular memories show an exceptional range of stability; depending on the strength of the feedbacks, cells may display memory of a previous expression state as short as a single generation to as far back as many thousands of generations [17]. However, quantitative measurements of phenotype stability, switching, and heritability are rare, both because detailed genealogical relationships are challenging to produce in single cells [36] and because reporters indicating degree of inheritance are not always available.

To measure how a dynamic gene expression state is inherited, we focused on an engineered version of the galactose utilization (GAL) pathway in the yeast Saccharomyces cerevisiae (Text S1). We disrupted the pathway's major negative feedback loop and grew cells in conditions where only a single positive-feedback loop was operational (see Materials and Methods). Under these conditions, cells stochastically transition between two distinct expression states even in the absence of an extracellular trigger. These infrequent switching events therefore likely arise from fluctuations in concentrations of regulatory proteins within the individual cells [37]. We are able to monitor transitions between ON and OFF using a fluorescent reporter (see Materials and Methods, Figure S3). Together, these attributes make our network an ideal model system that is well suited to study the heritability of an entire dynamic gene expression state.

In this work, we find that not only is the epigenetic phenotype itself heritable, but that the stability of this phenotype is likewise a heritable quantity. In other words, when cells divide, the nascent daughter cell assumes both the expression state of the mother cell as well as its tendency to switch epigenetic states at a similar time in the future. This is surprising, especially considering that individual cells viewed outside their genealogical context appear to switch completely at random. We resolve this apparent contradiction using a simple stochastic model.

## Results

### Heterogeneous Populations Are Generated from Single Progenitors that Spontaneously Switch between Two Phenotypes

We first set out to quantify, using fluorescence microscopy, the infrequent switching events that occur at random times. All experiments began with a single cell confined between a cover slip and a thick agar pad. Over a period of about 920 min (>15 h) each cell grew and divided to eventually form a small colony of 50–100 cells. Throughout the measurement period, these cells diverged in behavior, with some increasing in fluorescence and others decreasing. We repeated this process with more than 100 progenitor cells, so in sum our data represent many thousand single-cell trajectories.

**Figure 1:**
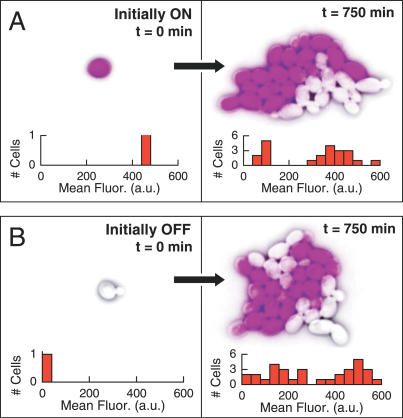
Cells Switch between Expressing and Nonexpressing States Images are phase contrast micrographs (black and white) overlaid with background-subtracted fluorescent signal (purple). (A) Over 750 min, or between 4 and 5 generations, an initially ON cell of strain MA0188 develops into a small variegated colony with subpopulations of ON and OFF cells. (B) An initially OFF cell likewise grows into a mixed colony with both ON and OFF cells. The sharp interface between ON and OFF cells in both (A,B) indicates that cell-cell communication does not play a major role in defining cell expression state. a.u., arbitary units.

We present two examples of the experimental procedure in Figure 1. In Figure 1A, an initially bright cell develops into a small colony with distinct subpopulations. The dim cells in the lower subpopulation continue to diminish in fluorescence with each successive cell division as the remaining molecules of green fluorescent protein (GFP) dilute. In Figure 1B, an initially faint cell likewise gives rise to a variegated colony with cells both dim and bright. Together, these two processes generate a broad bimodal steady-state distribution.

### Individual Cells Have Exponentially Distributed Switching Times

**Figure 2:**
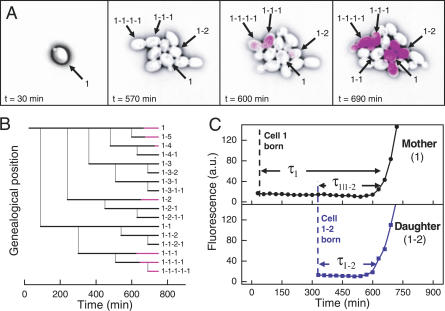
A Genealogical Switching History We designate the first cell in each movie cell 1 and sequential daughters of that cell 1–1, 1–2, 1–3. These daughter cells bud in turn, giving rise to cells 1-1-1, 1-1-2, 1-2-1, etc. (A) As in Figure 1, an initially OFF cell grows into a variegated micro-colony. Beginning at 600 min, or 4 generations, several cells fluoresce almost simultaneously. This includes the mother-daughter pairs (1,1–2) and (1-1-1,1-1-1–1). Conspicuously, cell 1–1 does not switch for the duration of our observation, even though its mother, daughter, and closest sibling all do. (B) The family tree for colony in (A). Black lines indicate cells in the OFF state, whereas pink lines represent cells after they have switched to the ON state. (C) Fluorescent time courses for mother cell 1 and her daughter 1–2, showing each as they switch into the ON state. The marginal switch times τ_1_ and τ_1–2_, run from cell birth until the beginning of the increase in fluorescence and do not depend on any other cells. The period labeled τ_1|1–2_ runs between the birth of cell 1–2 and the fluorescence of cell 1 and is an example of a conditional switch time.

Narrowing our focus to initially OFF progenitor cells, we allowed each to grow, divide, and give birth to other initially OFF cells. We then recorded instances when cells switched into the ON state (Figure 2A and Video S1). Because cellular auto-fluorescence is uniformly small throughout the population of OFF cells, these fluorescing events were generally distinguished unambiguously from background fluctuations. Using these data, we generated for each colony a family tree where the detailed genealogical relationships and gene-expression histories of corresponding family members are shown (Figure 2B).

**Figure 3:**
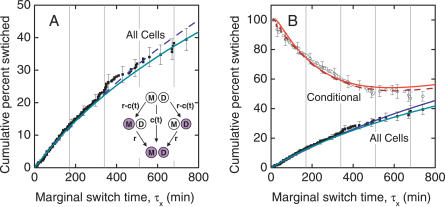
Single-Cell Fate (A) The cumulative percentage of cells that have switched is plotted against their marginal switch time. The black squares represent 251 switching cells, and the blue line is an exponential fit. The cyan dashed line is a result of our stochastic simulation (see Figure 5). Error bars are derived from a bootstrap analysis. The fits are consistent with the idea that a constant-rate process may underlie the network. The inset shows ways that mother-daughter pairs may switch, either dependently via the center route or independently of one another via the outer routes. (B) Gray circles describe the likelihood that a mother cell has switched given that its daughter cell is known to have switched before this time. The solid red line describes a two-parameter least-squares fit simultaneously to both curves with parameters described in the inset and main text. The dashed dark red line shows the fit resulting from the stochastic simulation. Black squares and blue lines are reproduced from (A) for comparison.

Because cells are continuously born throughout the experiment, we aligned them in silico so that their birth times were identical. In this context, it is natural to define the marginal switch time, τ*_X_*, a parameter that describes the interval between the birth of a cell *X* and the moment it eventually becomes fluorescent (Figure 2C). We normalized each measurement according to its expected likelihood of being observed (see Figures S4 and S5, Text S3) to account for any biases caused by the cells' exponentially dividing throughout our measurement period. The resulting data fit well to an exponential curve with an effective transition rate of 0.12 switches per generation (Figure 3A, cyan line). The slight discrepancy between data and exponential fit is likely the result of some cells growing out of the focal plane. The reverse switching distribution, composed of ON cells switching into the OFF state, could not be obtained in this simple way, because in this scenario the long life of the fluorescent proteins makes it difficult to determine the exact moment cells cease production of yellow fluorescent protein (YFP).

**Figure 4:**
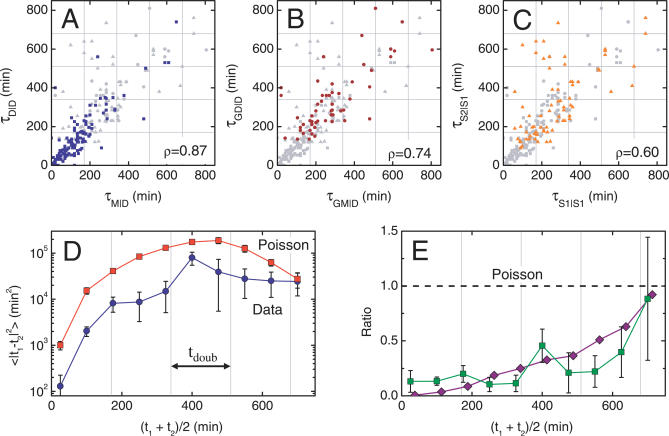
Cell-Pair Behavior The conditional switch times for closely related cells are compared. (A) The daughter switch time is compared to the mother switch times for 141 cell pairs. For times extending past 350 min (about two cell divisions), a strong correlation in times is observed. The other cell pair relationships, shown again in (B, C), are shadowed in grey. (B, C) The more distant relationships of GM-GD (*n* = 55) continue to show significant correlation, while the S1-S2 relationships (*n* = 74) shows somewhat less. The notable asymmetry of the S1-S2 distribution reflects the tendency for older siblings to sometimes switch before the younger sibling is even born. (D) In blue, the mean squared difference of the switch times from the combined relationships in (A–C), binned according to their average switch time. In red, a computer-generated Poisson simulation sets a bound for switching correlation in the limit of correlation tends to zero. The mean cell doubling time is labeled *t*_doub_. (E) Dark green squares show the ratio of the two curves in (D), demonstrating the persistence of a correlation for at least hundreds of minutes after cell division. In purple, the predicted fit from our stochastic simulation after fitting to the curves in Figure 3.

**Figure 5:**
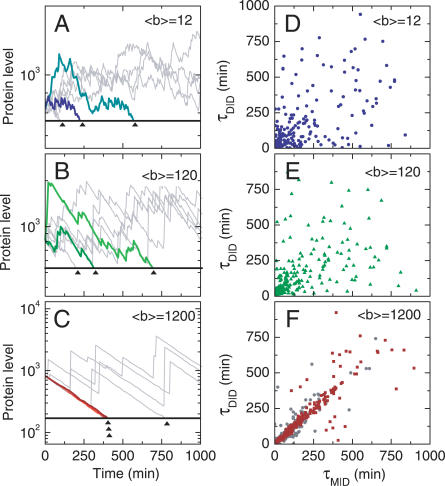
Burst-Induced Correlations Results of our stochastic simulation. (A–C) Fluctuations of protein concentration as a function of time are shown (gray lines). Several selected realizations are highlighted for emphasis. Proteins are created in bursts of size <b> = 12, 120, and 1,200 respectively for the three panels, with the average protein level held fixed. Thresholds (solid black lines) are chosen to result in an average switching rate equal to our experimentally measured value. When protein levels drop below a threshold, that cell is considered to have switched. (D–F) The resulting M-D scatter plots. As the burst size increases, the pattern becomes markedly more correlated. Gray circles in (F) are the experimental data reproduced from Figure 4A.

### Apparently Random Switches Are Heritable

This exponentially distributed switching pattern applies to cells chosen at random without regard to genealogy. However, measuring cells instead on the basis of their family history paints a very different picture. To demonstrate this difference, we asked how likely a mother and a daughter cell were to have both switched within a small window of time after the cells divided. We selected all daughter cells with marginal switch times below some value *T,* and then we measured what percent of their mothers had also switched at or before that time. The results, summarized in Figure 3B (open circles), show that when a daughter switches shortly after cell division, its mother cell is overwhelmingly likely to do the same. For example, of the daughters who switch within 400 min of cell division (about two generations), their mothers have approximately a 50% chance of switching in that same period. This represents a 2-fold increase in the switching rate for a typical unrelated cell. As *T* grows to encompass an ever-larger fraction of all daughter cells, the corresponding percent of switching mother cells asymptotically approaches the marginal switch distribution of Figure 3A (reproduced in black), which represents the limit of no genealogical relationship. As in the marginal switch case above, we are careful to weigh each of these mother-daughter pairs according to how likely we were to experimentally observe them.

To measure the underlying rates governing this process, we examined the possible switching events diagrammed in Figure 3A. In this simplified view, we assume cell pairs can either switch together into the ON state together at a rate *c(t)*, or independently of one another at a rate *r − c(t)*. In this way, the total switch rate for any given cell sums to *r* at all times, as required by the marginal switch distribution. We assume that the correlations decay with a rate 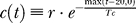 , which is reminiscent of an Ornstein-Uhlenbeck process (Figure 3A) [16,28]. The fixed delay of 20 min is included to account for slow chromophore (YFP) maturation as observed in our data (daughters that switch within the first 20 min after cell division have mothers that always switch). This model includes two free parameters: *r,* the overall switch rate, and *Tc,* the characteristic time for the correlation to decay. A global least-squares fit to both curves (Figure 3B, red and blue curves) simultaneously yields (*r* = [7.0 ± 0.5] · 10^−4^ min^−1^ = 0.12 ± 0.01 gen^−1^) and (*Tc* = 197 ± 54 min). This decorrelation rate is quite similar to the average cell doubling time of 177 min (Text S2 and Figure S1), and similar connections between doubling time and decorrelation have been found in other protein regulatory networks [28].

### Correlations of Switching Times between Cell Pairs Vary by Genealogical Relationship

The above analysis suggests that when cell pairs do switch, they will do so in synchrony. To demonstrate that this is indeed the case, we turned our focus to the further subset of cell pairs where both cells are observed to switch during the experiment (and therefore ignoring cases where only one cell in a pair switches). More specifically, we concentrated on three cell relationships: mothers with daughters (henceforth M-D), grandmothers with granddaughters (GM-GD), and older siblings with younger siblings (S1-S2). Instead of marginal switching times, which are measured relative to each individual cell's time of birth, we chose instead to compute the switch times of both cells relative to the moment when their two respective branches of the family tree first broke apart. Put another way, this quantifies the amount of time between a switching event and the last moment that these cell lines shared cytoplasm. The purpose of this approach is to allow us to compare cells that were born at very different times on equal footing, ensuring that switching events are measured relative to the same point for both cells. For M-D pairs, the time we use is simply the birth of the daughter; for GM-GD pairs, however, it is the birth of the intervening daughter; and for S1-S2 pairs, it is the older sibling's birth. Formally we define the conditional switch time, τ*_X_*_|*Y*_, as the time elapsed between the fluorescing of cell *X* and the birth of cell *Y*. When *X* and *Y* both refer to the same cell, we recover the marginal switch time (i.e., τ*_X_*_|*X*_ = τ*_X_*).

Comparing M-D conditional switch times (Figure 4A), we observe nearly synchronous switching that extends at least 300 min and yields a correlation coefficient of ρ_MD_=0.87 (*p* < 10^−45^). GM-GD and S1-S2 pairs (Figure 4B and 4C) show somewhat lower correlation coefficients of ρ_GMGD_ = 0.74 (*p* < 10^−9^) and ρ_SS_ = 0.60 (*p* < 10^−7^), respectively, although the total coefficient for all data combined remains a robust ρ_TOT_ = 0.8 (*p* < 10^−62^). The strength and duration of these correlations are surprising and were not found in bacterial [16,25] and mammalian [38] studies, except in context of morphological traits [39]. Like the marginal switch data, these scatter plots should be viewed in the context of finite experimental viewing times, giving weights to points that are inversely proportional to the number of experimental opportunities to have seen them (Text S3, Figures S6 and S7).

### Memory of Switching Persists for Several Generations

One dynamic measure for the randomness associated with the distribution is the average square difference of switch times for pairs of cells with comparable mean switch times (Figure 4D, blue curve). This curve rises rapidly at first, but at longer times it flattens out. This flattening is likely due, at least in part, to the limited duration of our experiments (on average 920 min), which constrains the scatter distribution to reside in the box shown in Figure 4A–4C.

To understand what this means, it is helpful to compare our results to those obtained using a stochastic Poisson model [40], where closely related cells are assumed to switch independently of one another and with constant probability in time (Text S3, Figures S2 and S8). To compare directly with our data, we ran the simulation for the same duration as our experiment and included all cell-pair relationships, giving the more complicated curve shown in Figure 4D (red curve).

The ratio of the data's mean square variation to that of the Poisson simulation (Figure 4E, green curve) is a measure for how correlated cells remain after a given period of time has passed. Points below a value of one (Figure 4E, dashed line) represent correlated switching behavior, whereas points above it would signify anticorrelated behavior. For over 600 min, the distribution remains distinctly sub-Poissonian. Only for the longest measured times are there indications that the cells switch independently of their history, and even this is with large uncertainty. Put another way, pairs of cells often remain on approximately the same trajectory for several cell divisions, even though cell growth has diluted many of the relevant proteins to a fraction of their original level.

### Stochastic Model

To examine our results at a microscopic level, we constructed a simple model that allows us to probe how the rich correlated switching dynamics arise from a simple regulatory network. Specifically, we asked whether the stochastic fluctuations of a single regulatory protein in our system could simultaneously explain the observed Poisson switching behavior that is expected for randomly selected individuals and subsequent long–timescale correlations. One key protein, Gal80p, functions to regulate the expression of all other genes in the network (Text S1). When it is present in the nucleus, Gal80p binds in a highly cooperative manner to the transcription factor Gal4p and represses the expression of Gal2p, Gal3p, and YFP (Volfson et al., for example, assume a Hill number of 8 between Gal4p and transcription at the *GAL1* promoter [33]).

Such high levels of cooperativity frequently give rise to steep transfer functions, which can result in switch-like behavior. This means that even a small decrease in the concentration of Gal80p can cause the transcription rate of downstream genes to increase dramatically from a very small basal rate to a large maximal rate. Once the downstream protein, Gal3p, begins to be produced, it will lead to sequestering of Gal80p to the cytoplasm, completing the feedback loop and causing the cell to completely switch from the OFF to the ON state.

We constructed a simple model that captures the essential properties of this process. In our cells, Gal80p is present in very low numbers, and we therefore account for the effects of stochastic production and degradation for this protein. Protein bursting invariably increases noise levels by amplifying rare events such as changes in promoter activation or mRNA creation and destruction [18,22,41]. We assumed that the burst-size distribution was exponential in shape with a mean consistent with the results of Bar-Even et al., who found an average of 1,200 proteins per burst [30]. We further assumed that the decay rate of the protein is dominated by dilution and therefore set by the division time of the cell. Finally, we included in our model a nonzero chromophore maturation time of 20 min, as observed in our data. To account for the cooperativity between Gal80p and Gal4p, we assumed that when Gal80p levels drop below a threshold value a cell rapidly activates gene expression and enters the ON fluorescent state.

In total, the model has only three parameters: (1) mean number of Gal80p molecules present per cell, (2) the switching threshold, and (3) the Gal80p burst size estimated from literature. We estimated the first two of these parameters by fitting the model to the marginal and conditional switching distributions shown in Figure 3B. Once the theoretical switching rates were fit to the experimental data, we asked if the model explained the highly correlated switching times observed between related cells. Without any additional fitting parameters, we predicted the mother (τ_M|D_) and daughter (τ_D|D_) conditional switching times (Figure 5F, brown squares) as well as their mean squared deviation 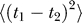 (Figure 4E, purple diamonds). These predictions matched remarkably well with the experimental data (Figure 4E, green boxes; Figure 5F, gray circles). The model therefore predicts that related cells will remain highly correlated in their switching times even though switching events seem to occur in a Poisson manner. A robustness analysis (Text S3, Figure S9) suggested a narrow range of possible values with an optimum centered around (average, threshold) ∼ (2,400 proteins, 670 proteins).

Bursting events in protein production are often associated with increases of noise in protein levels [18,22]. A counterintuitive aspect of our model is that the correlation observed in cell pairs comes as a consequence of stochastic bursting. As the burst size is ratcheted up from 12 to the experimentally observed value of 1200, for example, keeping average protein level and switch rate constant, correlations begin to emerge in the cell-cell scatter plots (see Figure 5). The reason for this effect is that the periods between bursting events are dominated by dilution of proteins, a relatively low-noise process. As the burst size is increased, the time between bursts must increase commensurately, leading to long periods of correlated behavior between cells. Two cells that start with the same amount of protein will therefore dilute that protein at a similar rate and switch ON (Figure 5C, black arrows) at similar times. Decorrelations can arise when one of the cells experiences a burst of new protein during this decay period. However, the cell experiencing the burst has a greatly reduced probability of switching ON in a short period of time. In this event, the cell will generally not be observed to switch at all over the duration of the experiment and consequently does not appear as a significantly decorrelated time-point in the τ_M|M_/τ_M|D_ scatter plot.

## Discussion

In recent years, cells within isogenic populations have become increasingly scrutinized as individuals, each with its own original behaviors and gene expression patterns. What make single cells distinctive, however, are not only the stochastic chemical reactions taking place within them but also their unique family histories. Here we have shown that a cell's decision to dramatically change expression states can hinge directly on this familial background. We have separated what, on its face, appears to be an exponentially distributed random process into stochastic and genealogically determined subcomponents. In addition, we show that protein or transcriptional bursting, which are processes that increase total noise in gene expression levels, can unexpectedly create correlated dynamic behavior between related cells, a phenomenon that would be lost in deterministic descriptions.

In the engineered network we used in this study, there is no reason to suppose that the correlations we observe provide an evolutionary advantage to cells. However, we can speculate that cells might use similar mechanisms to those we describe to coordinate behavior between themselves without relying on complex sensory machinery or physical proximity. Cells might exploit these architectures to ensure that when a switching event does occur, several other cells will do the same, effectively achieving strength in numbers. For example, a group of infectious disease–causing cells seeking to confront a host immune system might hypothetically choose to switch together from a slowly growing latent phenotype into an active virulent phenotype in a coordinated but randomly timed attack, thus enhancing their likelihood of sustaining an infection. Likewise, cells that benefit from cooperative metabolization could similarly benefit from temporally coordinated cooperation. It will be interesting to see how far similar analysis can be taken in the future and how many other systems might be found to have behavior so strongly influenced by family lineage.

## Materials and Methods

### Engineered destabilization of the GAL network.

We used the well-characterized GAL network as our model genetic network (see Text S1). In wild-type cells, transitions between the ON (galactose metabolizing) and OFF (unable to metabolize galactose) states is largely determined by the levels of inducers (e.g., galactose) or repressors (e.g., glucose) in the surrounding environment. To generate a switching phenotype with large dynamic range, we destabilized this in two ways. First, we removed the negative-feedback loop altogether by replacing the endogenous *GAL80* promoter with a weakly expressing, tetracycline-inducible one, P_TETO2_. Second, we grew the cells in the absence of galactose, which fully eliminates the *GAL2-*mediated positive feedback and weakens the *GAL3* feedback. Even in the absence of galactose, Gal3p has constitutive activity and, in sufficient quantities, can activate the network [42]. Considering the lower levels of Gal80p in our construct, this constitutive activity is likely a significant factor. Finally, the state of the network is read with P*_GAL1_*-YFP, with fluorescing cells considered ON.

Cells engineered in this way transition between ON and OFF states in a seemingly stochastic fashion. Cells with this genotype exhibit an extremely broad steady-state expression histogram, with fluorescence values that span more than two orders of magnitude, and the histogram has peaks on both the high and low expression limits, suggesting a bistable system with relatively infrequent transitions between the two states.

### Growth conditions.

Before imaging, cells were grown at low optical density overnight in a 30 °C shaker in synthetic dropout media with 2% raffinose as the sole carbon source. This neutral sugar is thought to neither actively repress nor induce the *GAL* genes [43]. We grew our cells in the absence of tetracycline, so levels of Gal80p were determined by the basal expression level of P_TETO2_. Approximately 12 h later, cells were harvested while still in exponential phase, spun down, and resuspended in synthetic defined (SD) media. Next, cells were transferred to a chamber consisting of a thick agar pad (composed of the appropriate dropout media and 4% agarose) sandwiched between a cover glass and slide. The high agarose density constrains cells to grow largely in a two-dimensional plane.

### Microscopy.

Fluorescent and phase-contrast images of growing cells were taken at intervals of 20–35 min on 10 different days for over 100 initial progenitor cells. Image collection was performed at room temperature (22 °C) using a Nikon TE-2000E inverted microscope with an automated state (Prior Scientific; http://www.prior.com) and a cooled back-thinned CCD camera (Micromax, Roper Scientific; http://www.roperscientific.com). Acquisition was performed with Metamorph (Universal Imaging; http://www.photomet.com).

## Supporting Information

Figure S1 Growth for Selected Cell Colony in Measurement Chamber Remains Constant for Longer than our Typical Measurement Period (17 KB PDF)

Figure S2 Histograms of Doubling Times as a Function of Previous Cell Divisions (10 KB PDF)

Figure S3 Rapid Maturation of YFP (21 KB PDF)

Figure S4 The Cumulative Percent of Cells That Have Switched Is Plotted against Their Marginal Switch Time (21 KB PDF)

Figure S5 Corrective Factors Used to Weigh Data Points According to Their Significance (15 KB PDF)

Figure S6 Schematic for Weighing the GM-GD Opportunity Windows and the Corresponding Window of Available Switch Times for the Example Family Tree (11 KB PDF)

Figure S7 Opportunity Windows for M-D, GM-GD, and S1-S2 Cell Pairs (22 KB PDF)

Figure S8 Overlay of Switching Patterns for all M-D, GM-GD, and S1-S2 Cell Pairs and Overlay of the Same in the Gillespie-Based Model (21 KB PDF)

Figure S9 Monte-Carlo Model Confidence Intervals (7 KB PDF)

Video S1 A Single OFF Cell Grows Over 690 min into a Small Colony of 16 Cells with Fluorescence Overlaid (6.3 MB AVI)

Text S1 Network Description (26 KB DOC)

Text S2 Additional Measurements (26 KB DOC)

Text S3 Data Analysis Methods (33 KB DOC)

## Abbreviations

GAL: galactose utilization
GM-GD: grandmothers with granddaughters
M-D: mothers with daughters, S1-S2, older siblings with younger siblings
