# Supplementary material for: Heritable Stochastic Switching Revealed by Single-Cell Genealogy
Source: PLoS Biol. 2007 Sep 4;5(9):e239. doi: 10.1371/journal.pbio.0050239 (PMC1964776; doi:10.1371/journal.pbio.0050239)

Supplementary Figure S9

Heritable Stochastic Switching Revealed by Single Cell Genealogy

Kaufmann, Yang, Mettetal, and van Oudenaarden

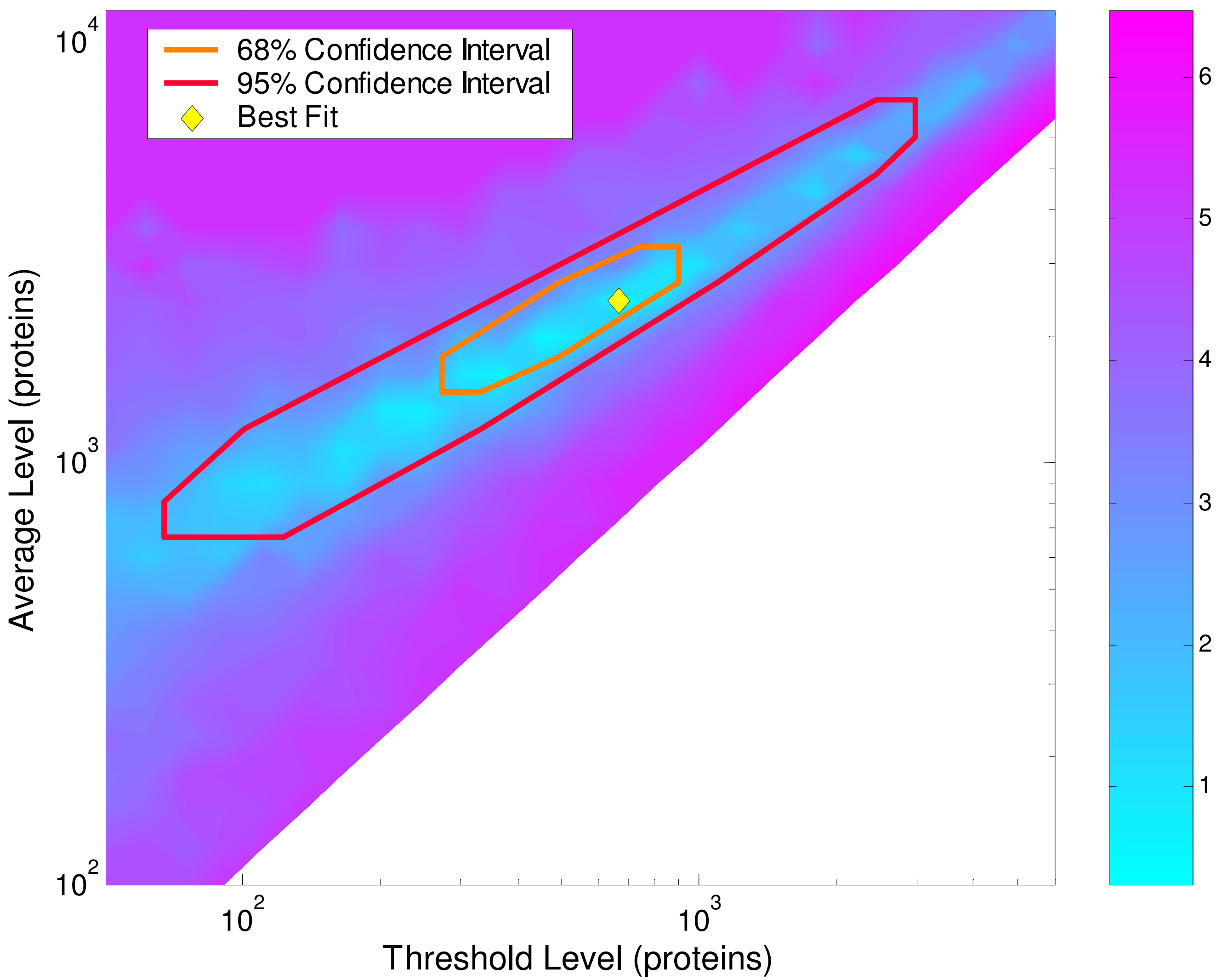

Supplement: Supplementary file 9 [file pbio.0050239.sg009.pdf]
