# Supplementary material for: Heritable Stochastic Switching Revealed by Single-Cell Genealogy
Source: PLoS Biol. 2007 Sep 4;5(9):e239. doi: 10.1371/journal.pbio.0050239 (PMC1964776; doi:10.1371/journal.pbio.0050239)
Supplement: Supplementary file 10 [file pbio.0050239.sd001.doc]

**Supplementary Text S1**

Heritable Stochastic Switching Revealed by Single Cell Genealogy

Kaufmann, Yang, Mettetal, and van Oudenaarden

**Network Description**

Elaborate regulatory machinery has evolved to help cells focus on the carbon sources that maximize their growth rate in a particular environment. The much studied galactose utilization network (GAL) is a model for such decision/metabolic pathways. The network, comprising roughly a dozen genes, contains two positive and one negative transcriptionally-mediated feedback loops nested one within the next. The positive loops make possible a two-state ‘all-or-nothing’ arrangement, while the negative loop is thought to stabilize cells in one of these two states, called ON and OFF.

The ON state’s defining characteristic is high activity of Gal4p, a transcriptional activator constitutively bound to the promoter of many GAL genes^1^. Gal4p activity occurs only in the absence of a dominant repressor Gal80p, which becomes sequestered to the cytoplasm in the presence of active Gal3p^2^. GAL3, in turn, is positively regulated by the level of active Gal4p, closing the first positive feedback. Thus Gal3 presents something of a chicken-or-the-egg situation: high expression of Gal3p leads to the activation of the GAL network, and activation of the GAL network leads to high expression of Gal3p.

A membrane protein Gal2p, whose expression is also regulated by Gal4p, forms a second positive feedback loop by importing galactose into the cell and consequently activating Gal3p. Finally, GAL80 negatively regulates its own production, again by suppressing the activity of Gal4p. For cells in the OFF state, the situation is reversed with low Gal4p activity, presence of repressor Gal80p at the GAL promoters, and a depletion of (active) Gal3p and Gal2p in the cytoplasm.

In wild-type cells, transitions between the OFF and ON states can be forced by changing levels of inducers (e.g., galactose) or repressors (e.g., glucose) in the surrounding environment. At intermediate levels of inducer, the OFF or ON status of the cell depends on the history of the media in which their ancestors grew, indicating significant hysteresis. This hysteretic behavior buffers against switching too rapidly between states, perhaps to avoid the metabolic cost incurred.

1. Keegan, L., Gill, G. & Ptashne, M. Separation of DNA binding from the transcription-activating function of a eukaryotic regulatory protein. Science 231, 699-704 (1986).

2. Peng, G. & Hopper, J. E. Gene activation by interaction of an inhibitor with a cytoplasmic signaling protein. Proc Natl Acad Sci U S A 99, 8548-53 (2002).
