# Supplementary material for: Heritable Stochastic Switching Revealed by Single-Cell Genealogy
Source: PLoS Biol. 2007 Sep 4;5(9):e239. doi: 10.1371/journal.pbio.0050239 (PMC1964776; doi:10.1371/journal.pbio.0050239)
Supplement: Supplementary file 11 [file pbio.0050239.sd002.doc]

**Supplementary Text S2**

Heritable Stochastic Switching Revealed by Single Cell Genealogy

Kaufmann, Yang, Mettetal, and van Oudenaarden

**Additional Measurements**

**S2.A Growth rate for colonies**

To calculate the effective bulk growth rate for the cell population, we measured the total area of micro-colonies as they grew from one cell to several hundred. This area grew exponentially with an average doubling time of 177 ± 24 min and remained constant over 24 hours (Figure S1), although slight day-to-day variations were observed (CV=0.14). For times longer than about 30-35 hours, the slide becomes confluent with cells. Typically we cease experiments after about 17 hours when cells become densely packed or begin to get pushed out of the focal plane and into the agar pad.

**S2.B Doubling times for individual measured cells**

As cells grew and divided, we recorded the times when each cell septated from its daughter cell. Newly born cells took on average 205 ± 58 minutes to give birth to their first daughter cell. Subsequent buddings, however, require only 157 ± 51 minutes for the subsequent 2-4 divisions, consistent with measurements others have made^1^.

**S2.C Demonstration of rapid fluorophore maturation**

Once the PGAL1–YFP gene becomes active, the delay until fluorescence is detected is limited by rates of transcription, translation and chromophore maturation. To measure the duration of this process, and in particular to be certain that slow YFP maturation was not the reason for the long correlations, we grew cells (MA182) with the endogenous GAL3 gene removed and replaced by a doxycycline-inducible one, tuned (at 0.05μg/ml dox) so that Gal3p levels were approximately the same as those in induced wild type cells. This strain was selected because the constitutive presence of Gal3p in the cytoplasm would enable the rapid activation of the GAL genes when galactose was added.

We first grew these cells overnight in media with doxycycline, 2% raffinose and no galactose. About 20 hours later, we spun down the cells and resuspended them in 2% raffinose and 1% galactose. Immediately we transferred the cells into our chamber and waited until cells settled in place (27 min). Then, at intervals of 15 minutes, we imaged their fluorescence. Between the first two planes, at 27 and 42 min respectively, we already see an increase in fluorescence, which from that point on continues to rise. A conservative estimate then of the time from activated PGAL1-YFP gene to observable signal is about 75 minutes, far less than the 177 minute doubling time of a typical cell.

The opposite transitions, from ON to OFF, are difficult to localize temporally because at the instant the cell goes into the OFF state fluorescence levels are still high. Only by diluting or degrading the YFP can the OFF state be ascertained. However, because doubling times, and therefore growth rates, vary from cell to cell, conclusively proving a cell’s network is OFF requires repeated measurements over long times. In contrast, switches from OFF to ON become rapidly apparent.

1.	Hartwell, L. H. & Unger, M. W. Unequal division in Saccharomyces cerevisiae and its implications for the control of cell division. *J Cell Biol* **75**, 422-35 (1977).
