# Supplementary material for: Heritable Stochastic Switching Revealed by Single-Cell Genealogy
Source: PLoS Biol. 2007 Sep 4;5(9):e239. doi: 10.1371/journal.pbio.0050239 (PMC1964776; doi:10.1371/journal.pbio.0050239)
Supplement: Supplementary file 12 [file pbio.0050239.sd003.doc]

**Supplementary Text S3**

Heritable Stochastic Switching Revealed by Single Cell Genealogy

Kaufmann, Yang, Mettetal, and van Oudenaarden

**Data Analysis**

**S3.A Corrections to the 1-D data required from finite viewing window**

The raw distribution of τ_X_ over our entire population (N=251 switched cells), excluding original progenitor cells because no birth time is known, fits an exponential (Figure S4, triangles and green line) with a time constant of 208 min. However, only 16% of cells were observed to switch. To interpret these results, we must account for the fact that cells exponentially divide throughout our measurement period. While this greatly expands the total number of observed cells, it inevitably means that some cells will be observed for longer periods than others. Thus very long switching events that span many cell generations, whenever they are observed, deserve special weight.

There are at least two ways to do this. The simplest way to correct for this variation in cell observation time is to measure the bulk growth rate (Figure S1). An exponential curve decaying with that time constant (Figure S5, red) approximates the relative likelihood of viewing a cell for a given duration. An exponential curve with that time constant is a reasonable estimate in the limit of a large number of division events (Figure S5, red). Applying this corrective factor to the marginal switch times provides an estimate of what the switch time distribution would look like had we collected data for an unlimited amount of time (Fig. 3A, squares). Further assuming that all cells eventually would switch, we fit the data to an exponential and arrive at an effective transition rate of 0.14 switches per generation (Fig. 3A, violet dashed). The slight discrepancy between data and exponential fit is likely the result of some cells growing out of the focal plane.

An alternative strategy is to use the actual doubling times for single cells. Drawing randomly from these distributions (N=361), we generate an ensemble of simulated family trees. To get the weighting factor for a switching event of length T, we count the number of cells in the simulated family trees that had times equal to or greater than T, and then divide that by the total number of cells. When very numerous (or very large) simulated family trees are used, this curve will closely approximate the exponential curve (Figure S5, black). However, as explained in the following section, this latter strategy has the advantage of being easily extended to two dimensions.

**S3.B Corrections to the 2-D scatter data required from finite viewing window**

Like the one dimensional marginal switch data, the 2-D scatter plots must also be viewed in the context of finite experimental viewing times. Because not all regions in the x-y plane are sampled with the same frequency, points from infrequently sampled regions must be given special emphasis. Drawing from the measured doubling times (N=361), we generated an ensemble of simulated family trees. An example of one of these family trees is shown below (Figure S6a).

In the figure, cell 1 (the GM) gives birth to cell 1-1 at time T_D_. Then, at time T_GD_, cell 1-1 in turn gives birth to 1-1-1 (the GD). The experiment then ends at some time Tend. In order to observe GM-GD switch pair (cells 1 and 1-1-1), each of these cells much switch before Tend. Cell 1 can switch anywhere in the range (T_D_,T_end_) [Note: if cell 1 switched before T_D_ then it gives birth to ON cells which, by our definition, cannot switch.] Cell 1-1-1, on the other hand, can switch in the range from its birth to the end of the experiment (T_GD_,T_end_). These two ranges define the rectangle of Figure S6b.

To generate the full weighing distribution for all points, the process is then repeated for all possible GM-GD pairs. The resulting rectangles are added together and the result is Figure S7. The inverse of these values is then used as the weighing factor. This strategy ensures that a pair of cells switching simultaneously in the microscope will always receive equal conditional switch times.

### S3.C Calculation of the cumulative percent switched

Our measurement of the cumulative percent switched (see Fig 3a main text) requires an estimate of the fraction of cells that switch within our measurement period. However, when the colonies grow to a large size, the cells crowd together and can sometimes be difficult to measure. We therefore focused our attention on all cells born at or before the sixth generation (e.g., cells 1-2, 1-1-1-1-1-1 and 1-3-1-1 were included, but 1-7 was not). In addition, we excluded all original progenitor cells (i.e., cell 1) because these cells may act differently as a result of having been spun down in the centrifuge prior to placement in our chamber (this condition changes our result by less than 2%). From this large subpopulation, which comprises most of the cells, we counted the percentage (15.8%) of observed switching cells.

### S3.D Calculation of the conditional percent switched

The conditional percent switched is calculated in the following way: First, we select all daughter cells that were found to switch before some time T. Second, we divide this population of daughter cells in to two groups, one with mothers who also switched at or before time T and another with mothers who switched after time T or did not switch. For each Mother-Daughter switch pair, a weight is assigned (as described Text S3) to take into account the likelihood of having viewed it. (In cases where the mother does not switch, the daughter’s switch time is used to generate this weight.) Next, the weights of all the cells in the two groups are added together. The conditional percent switched is defined as the sum of the weights of the cell pairs that switched before T, divided by the sum of all the weights of all cell pairs.

### S3.E Calculation of mean squared deviation

To generate the mean squared deviation of main text Fig. 4d, we first combined all observed MD, GMGD, and SS switch pairs (Figs. 4a-c) into a single data set. This was done to increase statistical power. These data sets consisted of two columns, $t_{1}$ and $t_{2}$, representing the older and younger cell switch times for 274 cell pairs. From this data set we used the bootstrap technique (draw with replacement) to generate 1000 alternate samples. Then, using each of these generated samples in turn, we transformed the data in the following way:

$$\begin{matrix} S=\frac{(t_{1}+t_{2})}{2} \\ D=|t_{1}-t_{2}|^{2} \end{matrix}$$

We next binned the transformed data according to $S$, using bins of sizes 25, 50, 100, and 150 min, with centers staggered by 75 min. At each center point, we calculated for all bootstrap samples and all bin sizes the average of the quantity $D$. Thus for each bin center we had four thousand estimates for the mean squared deviation (4 bin sizes times 1000 bootstraps). The mean and standard deviation of these four thousand estimates gives the mean and error shown on main text Fig. 4d.

### S3.F Generation of the Poisson model

In our MATLAB (MathWorks, Natick, MA) simulation, cells are made to virtually grow and divide according to the measured single-cell doubling times (Figure S2). These virtual cells are assumed to begin OFF and then allowed to switch to the ON state with constant probability. To calculate the wait time for these switches, we used the Gillespie algorithm^1^. Once a cell makes a switch, its time is recorded and the remaining OFF cells continue to divide in the simulation. Like data collection at the microscope, we run several hundred of these simulations, each for fixed periods of 920 simulation minutes (the median experimental run time). Our simulated data therefore contain the same ‘sampling artifacts’ (see Figure S7) as our measured data. The simulation produces switch data in the same form as the microscopy data, and is then processed identically (Figure S8). A similar scatter plot can be obtained by randomly drawing from the marginal switching distributions and in that way generating an uncorrelated scatter plot. This method is less accurate however because the resulting distribution will not be consistent with the opportunity windows described in Text S3.B (above).

**S3.F Confidence intervals for Monte Carlo model**

There are two free parameters in our stochastic model (see ‘Stochastic Model’ in main text): the average protein level and the switching threshold. Chi-squared values were computed for different combinations of these values, yielding a best-fit value of (average, threshold) ~ (2400 proteins, 670 proteins) and corresponding confidence intervals (see Figure S9).

1.	Gillespie, D. T. Exact Stochastic Simulation of Coupled Chemical Reactions. *The Journal of Physical Chemistry* **81**, 2340-2361 (1977).
